# Bioconversion of potato solid waste into antifungals and biopigments using *Streptomyces* spp.

**DOI:** 10.1371/journal.pone.0252113

**Published:** 2021-05-21

**Authors:** Heidi Schalchli, Emilio Hormazábal, Álvaro Astudillo, Gabriela Briceño, Olga Rubilar, María Cristina Diez

**Affiliations:** 1 Biotechnological Research Center Applied to the Environment (CIBAMA-BIOREN), Universidad de La Frontera, Temuco, Chile; 2 Chemical Engineering Department, Universidad de La Frontera, Temuco, Chile; 3 Chemical Sciences and Natural Resources Department, Universidad de La Frontera, Temuco, Chile; Tallinn University of Technology, ESTONIA

## Abstract

Potato waste was processed and used as a sole substrate for simultaneously producing antifungals and biopigments using *Streptomyces* spp. Out of three different *Streptomyces* isolates, strain SO6 stood out due to its ability to produce antifungals against economically important fungal phytopathogens and intracellular biopigments using potato waste powders without additional nutrients. This strain also showed the potential to secrete a broad range of enzymes for fermentation of eight sugars that could be involved in potato waste bioconversion. The results of the fermentation assay indicated that *Streptomyces* sp. strain SO6 degrades potato wastes during submerged fermentation, diminishing total dry weight and increasing reducing sugars from 0.3 to 3.6 mg·mL^−1^ and total proteins from 70.6 to 187.7 μg·mL^−1^. The results showed that *Streptomyces* strain SO6 was able to convert the potato waste into 0.96 mg·g^−1^ of diffusible antifungals and 1.75 mg·g^−1^ of reddish-purple biopigments. On the contrary, an absence of pigment production was observed during the fermentation of the commercial medium used as reference. According to our results, replacement of commercial culture media with available low-cost agroindustrial wastes for producing bioactive chemicals is a real opportunity to enhance the *Streptomyces* pigment production and antibiotic sustainability with cost-competitiveness. To our knowledge, this is the first report on the simultaneous production of biopigments and diffusible antifungal antibiotics produced by *Streptomyces* spp. using potato solid waste as the sole nutrient source.

## Introduction

The agri-food sector generates large amounts of wastes that require sustainable eco-friendly alternatives for utilization rather than waste disposal [1]. According to the Food and Agriculture Organization (FAO) [2], one-third of food produced for human consumption is lost every year or wasted throughout the supply chain (~1.3 billion tons). The generated wastes are a rich source of microbial nutrients (e.g., carbohydrates, proteins, minerals, and vitamins) that can be used for synthetizing valuable bioactive compounds [3]. These include enzymes, antibiotics, and biopigments that can be produced by submerged and/or solid fermentation of nutrient-rich wastes [3–5]. Studies on the replacement of traditional culture media with agro-industrial wastes demonstrated increments of up to 50% pigment production by fungi [6]. Compared with fungal pigments, most bacterial pigments are still at the research and development stage; thus, it is necessary to intensify the research on bacterial pigment production to make them available on the market [7].

Some of the recent reports on bacterial pigments using agro-industrial wastes highlighted the use of *Erwinia uredova*, *Planococcus* sp., and *Rhodopseudomonas faecalis*. These bacteria have demonstrated their potential in the production of carotenoid-type pigments using different agro-industrial wastes [8–10]. Moreover, *Chryseobacterium artocarpi* was able to synthesize yellowish-orange pigments using liquid pineapple waste [11]. To the best of our knowledge, there are very few reports related to the use of agro-industrial wastes to produce biopigments and antibiotics by Actinobacteria species.

Actinobacteria is a diverse taxonomic phylum that is widely distributed in both terrestrial and aquatic environments, where these microorganisms play an important role in the decomposition and recycling of materials [11]. Within the phylum Actinobacteria, microorganisms belonging to the genus *Streptomyces* stand out because of their morphological and physiological versatility. The *Streptomyces* spp. are Gram-positive bacteria well known for their ability to produce antibiotics, biopigments, and other metabolites with different applications in the food industry, medicine, and agriculture [12–15]. Antibiotics synthesized by *Streptomyces* spp. have been reported to control human pathogens, as well as economically important plant pathogens, including *Botrytis cinerea* [16] and *Fusarium oxysporum* [17,18]. Some *Streptomyces* spp. have also been highlighted due to their ability to produce pigments, including melanin, carotenoids, and actinorhodin-related blue biopigments using commercial media [19]. Some of these biopigments are important not only for their pigmentation but also their anticancer [20] and antibiotic activities [21].

Despite the great potential of the *Streptomyces* genus to produce antibiotics against a wide spectrum of pathogens and biopigments, there are few studies on the use agro-industrial wastes as growth support compared to commercial culture media. The production of oxytetracycline by some *Streptomyces* spp. in agricultural wastes with high protein and fiber contents has been reported [22]. Recently, Kalaiyarasi et al. [23] also reported the ability of a *Streptomyces* sp. to synthesize antibiotics using various agro-industrial wastes as solid substrate, including pineapple peel, wheat bran, apple pomace, rice bran, tapioca powder, orange peel, and green gram husk. The authors concluded that the presence of starch in the culture medium enhanced the production of antibiotics. In this way, the synthesis of bioactive compounds has been related to different parameters of the nutritional support, including carbon and nitrogen sources [24]. Long et al. [25] also reported a relationship between biopigment production by *Monascus ruber* and starch hydrolysis. The previous facts indicate that starch-rich wastes could be efficient nutrient sources for biopigment production.

Among the most abundant starch-rich wastes are discarded potatoes. The worldwide potato production was calculated to be over 370 million tons in 2019 [26]. Of this global potato production, it is estimated that approximately 30% is discarded and not used for human consumption [27], constituting a source of organic pollution since wet potato waste is prone to rapid microbial spoilage. Alternatives to loss of potato production and waste generation are required to avoid or minimize potato waste production [28] and/or the implementation of green strategies for the eco-friendly exploitation of waste biomass to obtain high-value-added products [27]. Discarded potatoes have the same nutritional components as those potato tubers suitable for human consumption, with basic differences in size, form, and damage in peel (e.g., cuts and blemishes). Traditionally, potato waste is used for producing low-value animal feed and fertilizer or used as the raw material of biogas, which leads to waste of abundant nutritive materials [29]. However, the nutrient content of discarded potato makes it an interesting byproduct for obtaining high-value-added products with different biotechnological applications [30], especially since the agro-industrial wastes are considered a key component in the circular bioeconomy [31].

In Chile, a high amount of discarded potato is generated each season according to the Oficina de Estudios y Políticas Agrarias de Chile (ODEPA) [32], which is directly eliminated to avoid decomposition and proliferation of plant diseases or, if possible, sold at a very low price (less than 1 USD per 50 kg bag) for animal feed. An alternative for valorizing this potato waste is its use as a nutritional source for producing antifungals and biopigments by *Streptomyces* spp., which could offer product diversification for farmers and environmental sustainability (S1 Fig).

Interestingly, a recent study on the use of lignocellulosic materials for the simultaneous production of carbohydrates, lipids, and pigments by the anoxygenic photosynthetic bacteria *R*. *faecalis* was reported by Saejung and Sanusan [33]. These studies are a starting point for the further simultaneous production of microbial value-added products using agro-industrial wastes as low-cost nutrient sources for large-scale industrial production. To the best of our knowledge, there are no reports of the use of starch-rich wastes for the simultaneous production of antifungals and biopigments by *Streptomyces* spp. In this research, we aimed to evaluate the use of potato solid waste as a nutrient source for the simultaneous production of antifungal antibiotics and biopigments by *Streptomyces* spp. under submerged fermentation.

## Material and methods

### Chemicals and culture media

All solvents of chromatographic grade were purchased from Merck (Darmstadt, Germany): hexane (Hex), chloroform (Chl), methanol (MeOH), and ethyl acetate (EtOAc). Silica gel 60 (0.063–0.200 mm) for column chromatography (CC) and silica gel 60 F254 plates (20 × 10 cm) were also purchased from Merck (Darmstadt, Germany).

The culture medium potato dextrose agar (PDA) for fungus maintenance and antifungal assays was purchased from Merck (Darmstadt, Germany). The International *Streptomyces* Medium No. 2 (ISP2; glucose 4 g, yeast extract 4 g, malt extract 10 g, distilled water 1 L, pH 7.0) was used for propagation of *Streptomyces* strains. The previous ISP2 broth was jellified with agar-agar granulated (15 g·L^−1^) to prepare ISP2 agar medium. All the ISP2 components were purchased from Merck (Darmstadt, Germany).

### Potato waste processing

**Fig 1:**
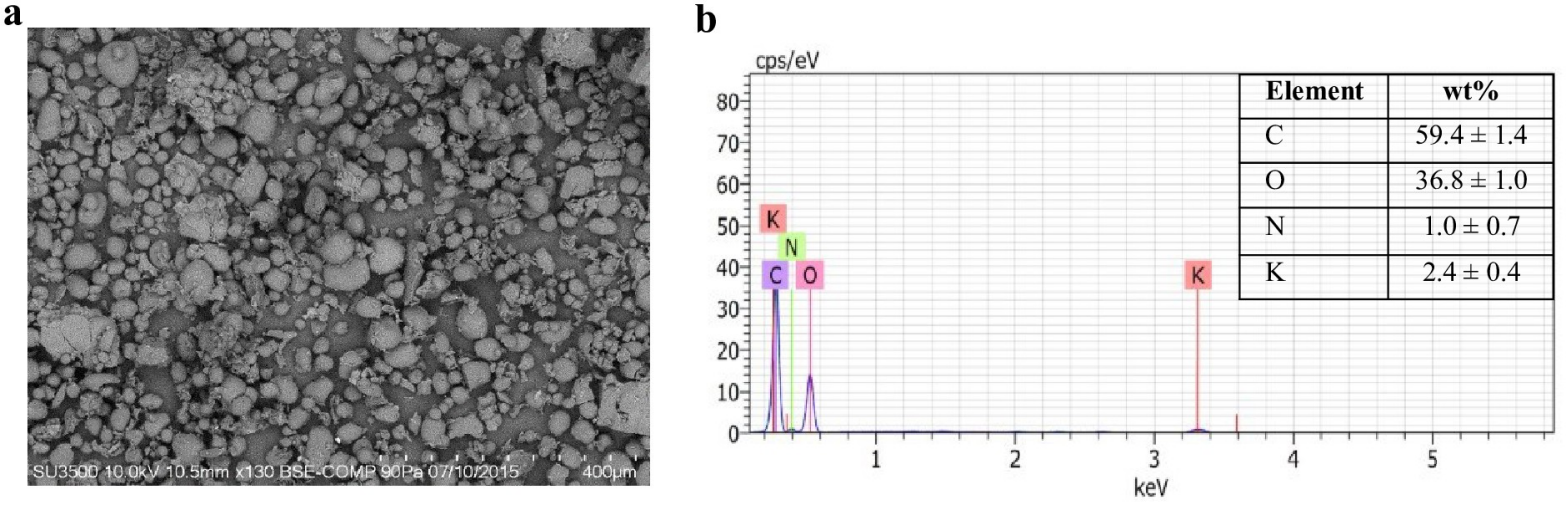
SEM image (a) and SEM-EDX (X-ray spectroscopy) spectrum (b) for the potato waste powder.

The potatoes (*Solanum tuberosum* L.) used in the preparation of culture media were discarded potatoes obtained from a local potato-producing company near Temuco City in Chile. These potatoes were cut into small pieces (1 × 1 cm), dried at 70°C for 24 h, and ground to a fine powder. The proximate composition of the potato waste powder (PW) was reported in our previous study, as described by Schalchli et al. [34] (moisture 7.8%; fat 6.2%; protein 7.9%; crude fiber 0.4%; ash 3.9; N-free extract 73.7%). In this study, a morphological and semiquantitative determination of the elemental composition of the PW surfaces was carried out by means of a variable pressure scanning electron microscope (VP-SEM, Model SU 3500-Hitachi; Hitachi Corp., Japan) with an X-ray spectroscopy (EDX) detector (Quantax-Bruker). The particle size of PW was between 10 and 200 μm (Fig 1a) and contained different proportions of carbon, oxygen, nitrogen, and potassium (Fig 1b). The PW was used to prepare PW broth (16 g·L^−1^) and PW agar (PW 8 g·L^−1^, agar-agar 15 g·L^−1^) without the addition of supplements and pH adjustment [34].

### Microbial strains

Three *Streptomyces* strains with different morphological characteristics and pigmentation were obtained from the strain collection at the Laboratory of Biotechnology, Universidad de La Frontera (Chile). The strains were previously isolated from soil samples and preserved due to their antifungal activity against *Mucor miehei*(Cooney et Emerson Tü 284). The *Streptomyces* strains SO6, PM1, and PM7 are conserved in the Chilean Culture Collection CCCT-UFRO with the following codes: CCCT21.05, CCCT21.03, and CCCT 21.04, respectively. For the assays, the bacterial colonies were maintained on ISP2 agar slants and stored at 4°C. The spore solution of *Streptomyces* spp. was prepared by flooding agar ISP2 plates with 20 mL of distilled water and used as inoculum at a spore concentration of 10^4^ spores·mL^−1^. A pre-inoculum for fermentation assay was prepared, consisting of 10^4^ spores·mL^−1^ of *Streptomyces* sp. SO6 cultured in 200 mL of ISP2 broth and incubated at 28°C and 120 rpm for 48 h in darkness.

The phytopathogenic fungi *B*. *cinerea* (CCCT21.01) and *F*. *oxysporum* (CCCT 21.02) were used as target fungi for evaluating the antifungal activity of *Streptomyces* strains. The fungus *M*. *miehei* (Cooney et Emerson Tü 284) was used as a reference strain for detecting antibiotic metabolites. For the assays, all fungal strains were maintained on PDA slants at 4°C. Before their use, the fungal strains were subcultured from stock slants onto PDA plates and incubated for 5 days at 25 ± 1°C. A spore solution of *B*. *cinerea* was prepared by flooding PDA plates with 20 mL of distilled water and used in disc agar-diffusion testing at a spore concentration of 10^6^ spores·mL^−1^.

### Characterization of *Streptomyces* spp.

The *Streptomyces* strains SO6, PM1, and PM7 were characterized in terms of morphological, biochemical, and molecular properties. To determine the morphological characteristics, samples of 5-day old *Streptomyces* cultures were analyzed using a VP-SEM (Model SU 3500-Hitachi; Hitachi Corp., Japan). For biochemical characterization, we used Api-Coryne test strips (Biomerieux^®^, USA) according to the manufacturer’s instructions. The amylase and protease activities were determined following the methodologies described by Minotto et al. [35] and Vaithanomsat et al. [36]. Lastly, the *Streptomyces* strains were characterized using a genetic affiliation analysis of 16S ribosomal DNA (rDNA) gene fragments from the *Streptomyces* genus. For this, the F341/R534 primer pair was employed [37]. The 16S rDNA fragments were sequenced in both directions using an automated sequencing system (Macrogen, Korea). Sequences were compared with the Basic Local Alignment Search Tool (BLAST) database.

### Evaluation of antifungal activity and biopigment production

The three *Streptomyces* strains were assayed for potential antifungal activity against the target fungi *B*. *cinerea*, *F*. *oxysporum*, and *M*. *miehei* using a dual culture assay on 90 mm PDA and PW agar (8 g·L^−1^ PW) plates according to Landa et al. [38], with modifications. Fresh cultures of 5-day old *Streptomyces* strains were streaked (approximately 4 cm line) on one side of the plate and incubated for 5 days at 28°C. Then, the plates were inoculated with one mycelial plug (6 mm in diameter) of 5-day old target fungi on the opposite side and incubated until the control plate reached the edge. Three replicate plates were used for each dual culture. The antifungal activity was evaluated by measuring the inhibition zones expressed in mm of inhibition against the target fungi according to Kim et al. [39]. An optical microscope (Euromex IS 1153-EPL model, Holland) was used to facilitate the localization of fungal hyphae and to avoid any subjective measurements of fungal growth inhibition.

The three *Streptomyces* spp. were cultured in PW broth at a concentration of 16 g·L^−1^ PW and ISP2 medium. For this, 1 L Erlenmeyer flasks containing 500 mL of each culture medium inoculated with a bacterial suspension of 10^4^ spores·mL^−1^ were incubated at 28°C and 120 rpm for 10 days on a rotary shaker in darkness. The pigment formation was confirmed by observation of colored cultures. Lastly, the strain able to inhibit all the target fungi and produce biopigments using PW as the sole nutrient source was selected for the batch fermentation assay.

### Batch fermentation assay

The batch fermentation assay was performed according to Sharmila et al. [40], with modifications. Briefly, 250 mL Erlenmeyer flasks containing 100 mL of PW broth (16 g·L^−1^ PW) without pH adjustment (initial pH ~6.2) were inoculated with fresh inoculum of *Streptomyces* sp. SO6 (4% *v*/*v*). The flasks were incubated at 28°C and 120 rpm for 10 days in darkness. Additionally, ISP2 medium was used for comparative purposes and incubated in the same conditions. The control treatment was the same PW broth and ISP2 medium without bacterial inoculum. Each assay was carried out in triplicate under destructive sampling mode. Every 24 h, the pH value using a pH-meter, reducing sugars using the DNS method [41], and total protein [42] were measured. For dry weight determination, the bacterial/PW biomass was separated by filtration and then dried at 105°C until a constant weight was achieved.

### Obtention of crude extracts

*Streptomyces* sp. strain SO6 was cultured in PW broth medium to obtain EtOAc crude extracts as previously described [40,43]. Two Erlenmeyer flasks (1 L) containing 500 mL of PW broth (16 g·L^−1^ PW) without pH adjustment (initial pH 6.2) were inoculated with a suspension of 10^4^ spores·mL^−1^ of *Streptomyces* sp. strain SO6 and incubated at 28 ± 1°C under agitation (120 rpm). After 2 weeks of fermentation, the culture was separated by vacuum filtration to obtain cell-free supernatant and bacterial/PW biomass.

The cell-free supernatant was extracted exhaustively with EtOAc, filtered in a decanting funnel, concentrated to dryness in a SPD121P SpeedVac^®^ Concentrator (Thermo Scientific Savant^®^), and stored at 4°C [44]. The biomass was maintained in 200 mL of EtOAc for 1 week at 4°C to macerate before final extraction. After this, the pigmented EtOAc was filtered, concentrated to dryness, and stored at 4°C. The ultraviolet/visible light (UV/Vis) absorption spectrum (380–750 nm) of pigmented EtOAc was determined using a Synergy H1 multimode microplate reader (BioTek) before conservation.

The obtained EtOAc crude extracts from cell-free supernatant and biomass were assessed separately for determining their antifungal activity using disc agar-diffusion testing [45] against the plant pathogenic fungus *B*. *cinerea* at a concentration of 100 μg·disc^−1^. For this, 100 mL of PDA medium was inoculated with *B*. *cinerea* (10^6^ spores·mL^−1^) and placed in 90 mm Petri dishes. Then, 100 μg of crude EtOAc extract previously solubilized in MeOH was placed on filter paper discs (6 mm in diameter) and deposited on the surface of the *B*. *cinerea* plate. After 4 days of incubation at 28°C, the inhibition zones were measured (mm in diameter).

### Isolation and purification of antifungals

The EtOAc crude extracts from cell-free supernatant were analyzed by CC combined with preparative and analytical thin-layer chromatography (TLC) to isolate fractions according to Siddharth and Rai [44], with modifications. For this, the crude EtOAc extract (~330 mg) was loaded onto a silica gel column (30 × 2.5 cm) and eluted with a gradient of solvents Hex/Chl/EtOAc/MeOH (100% Hex→100% MeOH). The fractions were collected and validated by TLC using silica gel 60 F_254_ plates and Hex:EtOAc:MeOH (7:2:1) as the mobile phase. The fractions with the same retention factor (*R*_*f*_) values, visualized with visible and UV light at 365 and 254 nm, were combined and concentrated to obtain homogeneous fraction groups. Two TLC plates were used in parallel in each experiment. Each fraction group was concentrated to dryness and analyzed for its antifungal activity against *B*. *cinerea* using the previously described disc agar-diffusion testing to identify the active fractions.

### Isolation and purification of biopigments

The EtOAc crude extract from the biomass was analyzed by CC combined with preparative and analytical TLC to determine its content of biopigments according to Siddharth and Rai [44], with modifications. The EtOAc extract (~58 mg) was loaded onto a silica gel column (30 × 2.5 cm) and eluted with a gradient of Hex/Chl/EtOAc/MeOH (100% Hex→100% MeOH). The fractions (63) were collected and validated by TLC using silica gel 60 F_254_ plates and Hex:EtOAc:MeOH (7:2:1) as the mobile phase. The fractions with the same *R*_*f*_ values, visualized with visible and UV light at 365 and 254 nm, were combined and concentrated to obtain homogeneous fraction groups. Two TLC plates were used in parallel in each experiment. On the basis of the presence of pigmentation with similar *R*_*f*_ values, the fraction groups F3 and F4 were pooled and analyzed by preparative TCL to determine their purity. The preparative TLC was carried out using silica gel 60 F_254_ plates and Hex:EtOAc:MeOH (7:2:1) as the mobile phase.

### Statistical analysis

Data from the dual culture assay (mm) were averaged, and the standard errors (SD) of the means were calculated. Differences among treatments were assessed with one-way analysis of variance (ANOVA) and post hoc analysis of differences in means was conducted with the Tukey test using JMP 11.0 software (SAS Institute Inc., NC, USA) with statistical significance set at *p* < 0.05.

## Results

### Characterization of *Streptomyces* strains

Differences in terms of the type of branch, colony pigmentation, gelatin hydrolysis, carbohydrate fermentation (glucose, ribose, xylose, and mannitol), and enzymes (*N*-acetyl-β-glucosaminidase and urease) were found between the active strains. The morphological and biochemical characteristics of the active strains are shown in Table 1. Analysis of the 16S rRNA gene revealed that the PM1, PM7, and SO6 strains show high levels of sequence similarity to *Streptomyces* spp. accession numbers EU216727 (93%), GU985264 (99%), and HM125709 (99%), respectively.

**Table 1:** Morphological and biochemical characteristics differentiating *Streptomyces* strains PM1, PM7, and SO6.

Characteristics	Actinobacteria strains		
	PM1	PM7	SO6
**Gram**	+	+	+
**Surface**	Rugose	Rugose	Rugose
**Type of branch**	Straight to flexuous	Open loop	Straight to flexuous
**Colony pigmentation**			
Aerial mycelium	Gray	Gray	Gray
Substrate mycelium	Dark red	Blue	Orange
Exopigment	+	+	-
**Nitrate reduction**	+	+	+
**Pyrazinamidase**	+	+	-
**Pyrrolidonyl arylamidase**	+	+	+
**Alkaline phosphatase**	+	+	+
**β-Glucuronidase**	-	-	-
**β-Galactosidase**	+	+	+
**α-Glucosidase**	+	+	+
**N-Acetyl-β-glucosaminidase**	+	-	-
**Urease**	+	-	+
**Esculin (β -glucosidase)**	+	+	+
**Gelatine hydrolysis**	+	-	-
*Amylolytic activity	+	+	+
*Proteolytic activity	+	+	+
**Fermentation of:**			
Glucose	-	+	+
Ribose	-	+	+
Xylose	-	+	+
Mannitol	-	+	+
Maltose	+	+	+
Lactose	+	+	+
Sucrose	+	+	+
Glycogen	+	+	+

(+) positive; (−) negative. The type of branch was determined by variable pressure scanning electron microscopy. Pigment color was determined in commercial potato dextrose agar (PDA) medium. Biochemical characteristics were determined using the ApiCoryne kit.

*Methodologies described by Minotto et al. [35] and Vaithanomsat et al. [36].

### Antifungal activity and biopigment production by *Streptomyces* spp.

The *Streptomyces* strains PM1, PM7, and SO6 were able to produce different culture pigmentations and inhibit the mycelial growth of the reference fungus *M*. *miehei* and the plant pathogenic fungus *B*. *cinerea* using commercial culture media. *F*. *oxysporum* growth was slightly inhibited by the PM1 and SO6 strains, but was not inhibited by PM7 (Table 2).

**Table 2:** Antifungal activity of *Streptomyces* strains against fungal plant pathogens and the reference fungus *Mucor miehei* (zone of inhibition in mm) using commercial and potato waste media as nutrient sources.

Strain	Inhibition zone of micelial growth (mm)					
	PDA medium			PW agar medium		
	*F*. *oxysporum*	*B*. *cinerea*	*M*. *miehei*	*F*. *oxysporum*	*B*. *cinerea*	*M*. *miehei*
**PM1**	5.0 ± 1.0	16.7 ± 0.6*	26.7 ± 1.5*	-	16.0 ± 1.0*	11.3 ± 1.5
**PM7**	-	2.7 ± 0.6	6.2 ± 0.8	-	-	13.3 ± 3.5
**SO6**	**3.7 ± 0.4**	15.3 ± 0.6*	**18.0 ± 1.0**	**5.3 ± 0.8**	**5.7 ± 1.2**	19.0 ± 1.7*

(-) not inhibited; PDA, potato dextrose agar (Merck); PW, potato waste.

Values are means ± SD (*n* = 3). Significant differences between strains within each plant pathogenic fungus are indicated by asterisks (*, *p* ≤ 0.05).

**Fig 2:**
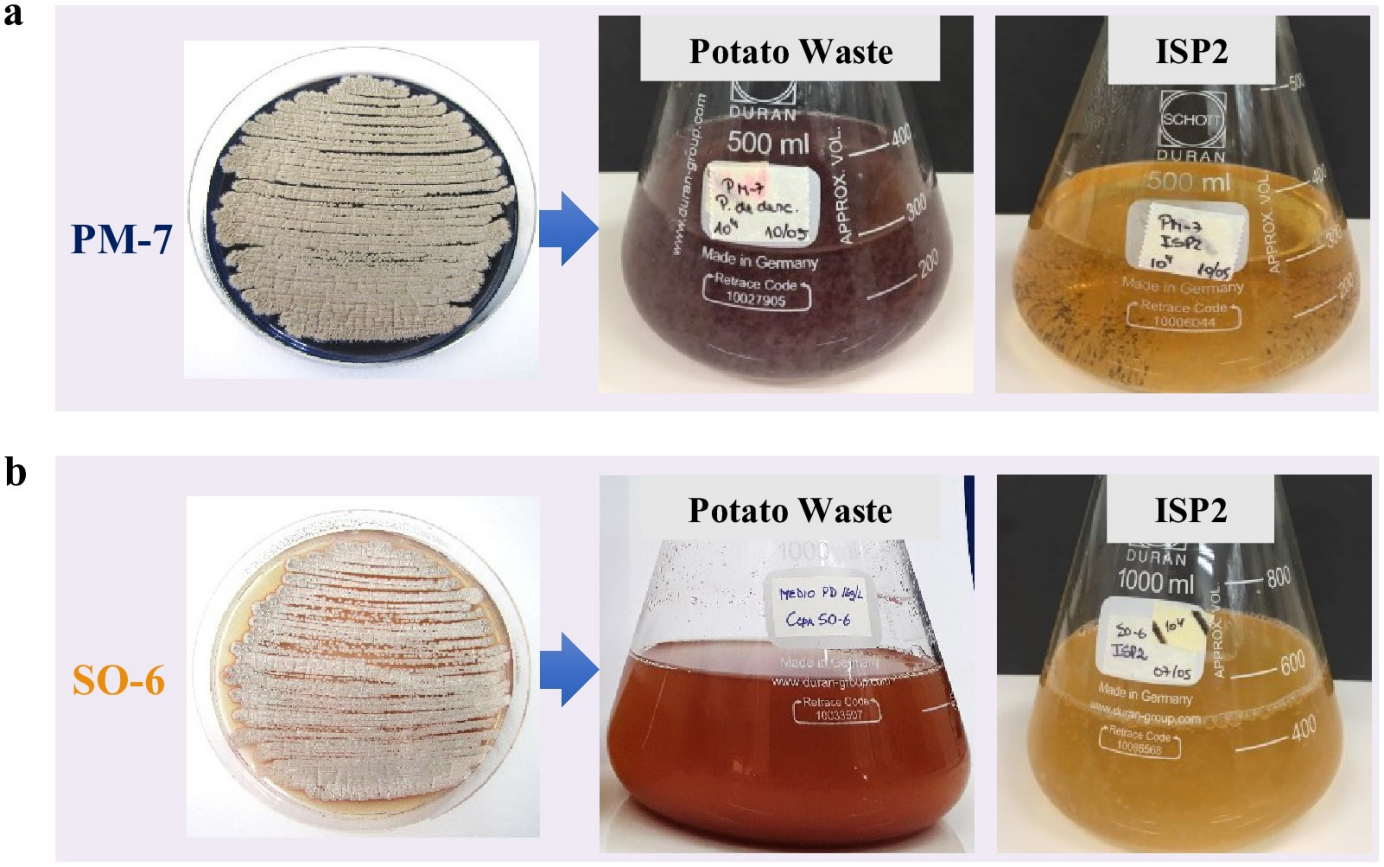
Pigment production by *Streptomyces* strains (10^4^ spores·L^−1^) using potato waste powder (PW) at a concentration of 16 g·L^−1^ and International *Streptomyces* Medium No. 2 (ISP2) incubated for 10 days. The strain PM1 was not able to produce pigments using PW as the sole nutrient source.

There were significant differences (*p* ≤ 0.05) among the antifungal activities of the three actives *Streptomyces* strains against the target fungi using both PDA and agar PW media. Concerning biopigment production, the strains PM7 and SO6 maintained their biopigment production when ISP2 medium was replaced with PW broth (Fig 2).

Although all the tested *Streptomyces* spp. were able to produce biopigments and inhibit *B*. *cinerea* and *M*. *miehei* growth using commercial media, the SO6 strain stood out due to its ability to produce antifungals against the three target fungi while maintaining its pigment production in the culture medium formulated with PW. Therefore, *Streptomyces* sp. strain SO6 was selected for PW fermentation assays and studies on the obtained bioproducts.

### Batch fermentation assay

**Fig 3:**
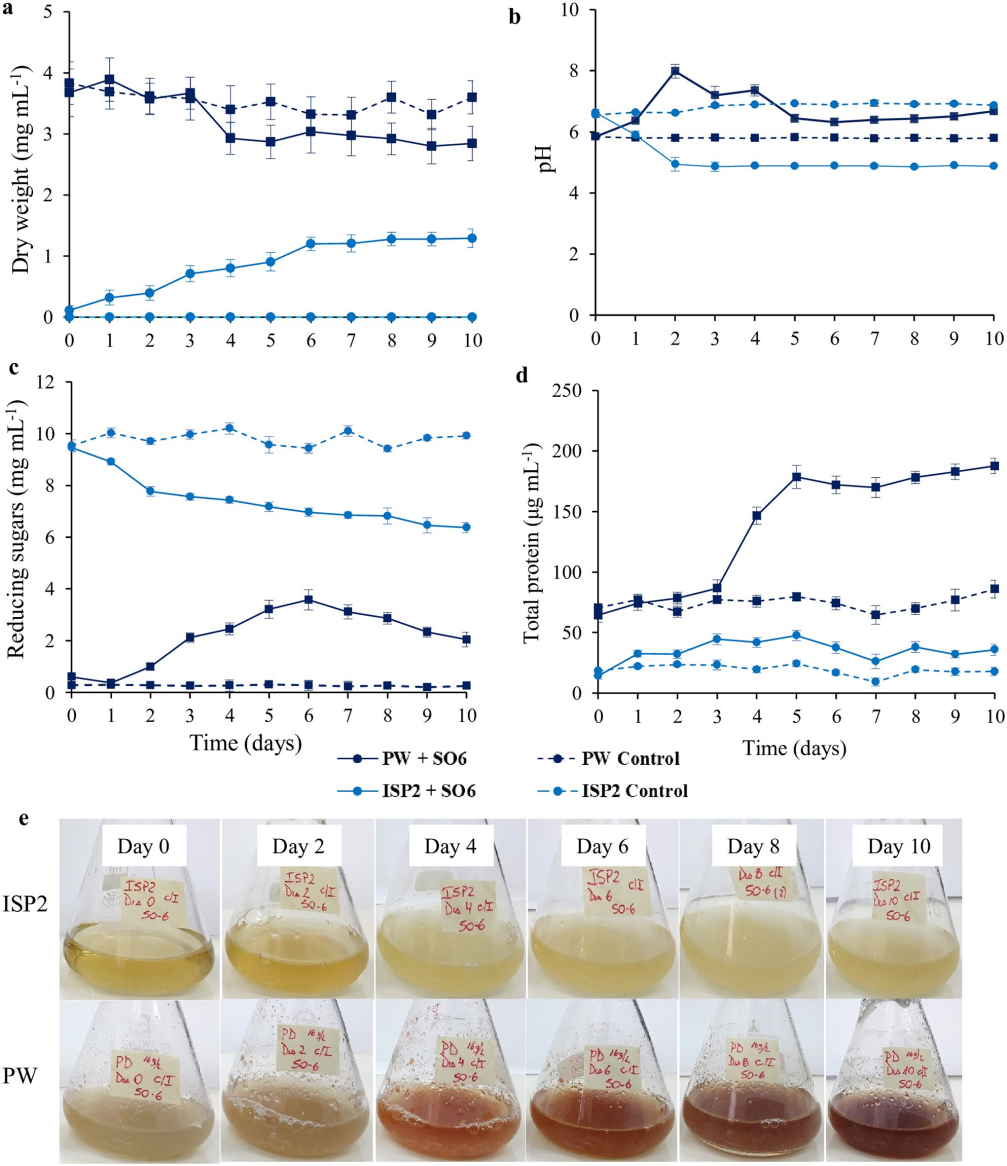
Dry weight (a), pH (b), reducing sugars (c), total protein (d), and pigment production (e) of *Streptomyces* sp. strain SO6 (inoculum 4% *v*/*v*) under submerged fermentation using potato waste powder (PW) broth (16 g·L^−1^) and ISP2 medium.

To test the potential use of PW as a nutrient source for producing high-value-added bioproducts by *Streptomyces* sp. strain SO6, submerged fermentation assays without pH adjustment and/or supplementation were performed. Fig 3 shows the dry weight of total biomass, pH, and reducing sugar and total protein content in PW broth and ISP2 (control treatment) over the 10 day fermentation assay.

As shown in Fig 3a, the dry weight increased during the first 6 days in ISP2 medium (log phase) and then remained constant at ~1.2 mg dry wt·mL^−1^ until the end of the fermentation assay (stationary phase). However, when the same *Streptomyces* sp. strain SO6 was grown in PW medium, the dry weight (PW and bacterial biomass) did not differ significantly (*p* < 0.05) from the control treatment (without bacteria) over the fermentation time. The PW broth with or without inoculum showed a higher dry weight than ISP2 treatments due to the particle size and solubility of the culture components. The pH decreased during fermentation of *Streptomyces* sp. strain SO6 into ISP2 medium from 6.6 to 4.9 (at day 2) and then remained constant until the end of the fermentation assay (Fig 3b). In contrast, the pH of PW medium increased considerably on the second day of incubation (from 5.8 to 8.0), remaining constant from the fifth day (~6.4). In addition to the microbial biomass increase in ISP2 medium, the content of reducing sugars decreased steadily from 9.5 to 6.4 mg·mL^−1^ at day 10. The initial reducing sugar content in PW medium was 0.6 mg·mL^−1^, which increased to a maximum of 3.6 mg·mL^−1^ on the sixth day of incubation. After the sixth day, reducing sugars consistently decreased until the end of the PW fermentation assay (Fig 3c). The changes in total proteins in both ISP2 and PW media are shown in Fig 3d. The maximum value of total protein content in ISP2 medium with *Streptomyces* sp. strain SO6 was 47 μg·mL^−1^ on the fifth day of incubation, remaining constant over time (Fig 3d). Interestingly, total protein secretion was noticeably higher in PW inoculated with *Streptomyces* sp. strain SO6 compared to the control flask, reaching 179 μg·mL^−1^ of total proteins on the fifth day of incubation (~3.8-fold increase).

Lastly, pigment formation was observed in PW medium on the fourth day of incubation, whereas there was no pigmentation in ISP2 over the time considered for the fermentation assay (Fig 3e). The UV/Vis spectra revealed a maximum absorption peak (λ_max_) at 520 nm. A relationship between the pigment concentration and the PW concentration over time was also observed (result not shown).

### Antifungal and biopigment production by *Streptomyces* sp. strain SO6

**Fig 4:**
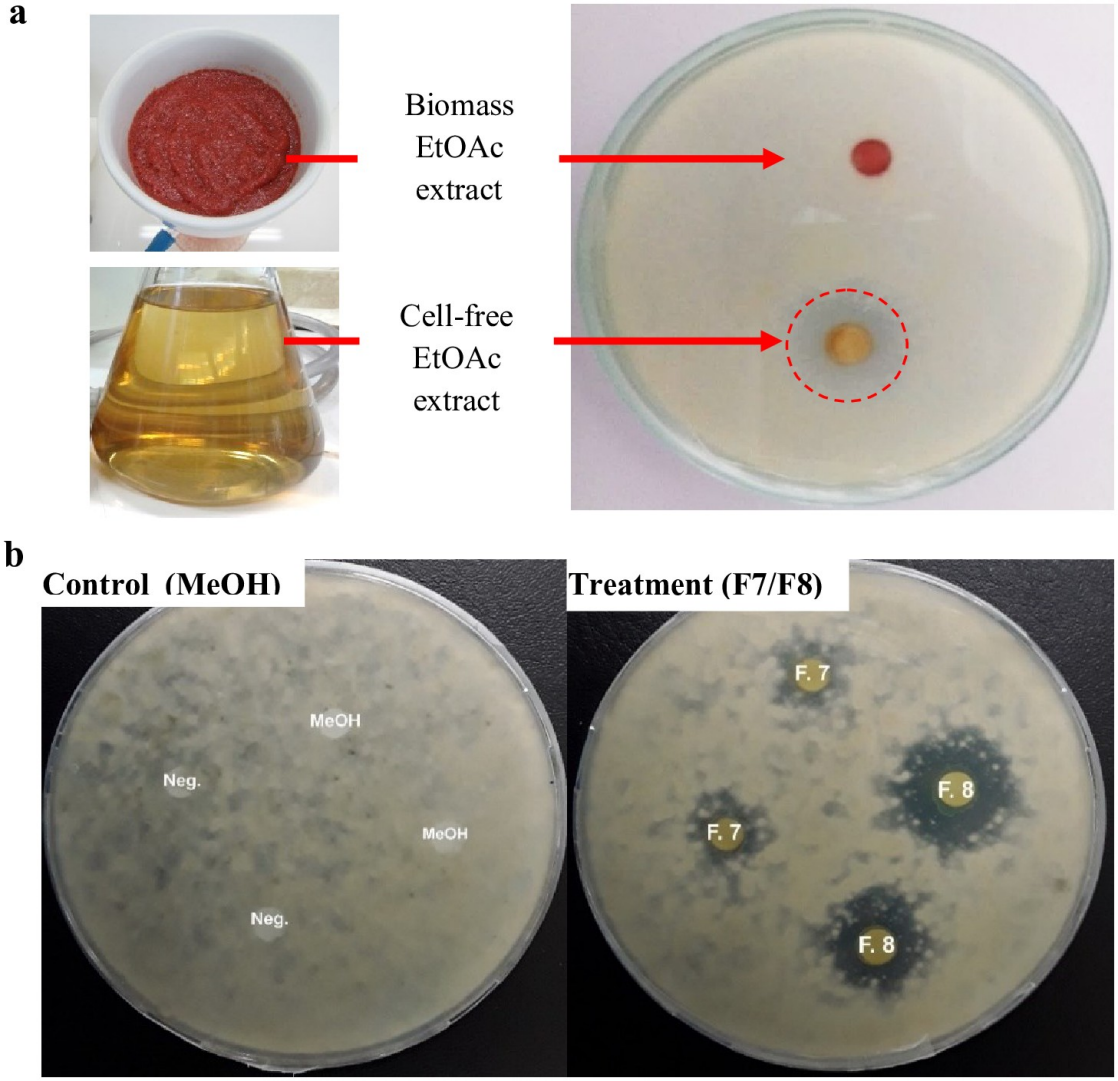
Antifungal activity of crude EtOAc extracts (a) and active fractions of cell-free EtOAc extracts (b) produced by *Streptomyces* sp. SO6 against *Botrytis cinerea* (10^6^ spores·mL^−1^). Plates were incubated for 4 h at 28 ± 1°C. The red circle indicates the inhibition zone (19 mm in diameter).

An initial analysis of the antifungal activity of the EtOAc extracts from both cell-free supernatant and biomass was performed to identify active extracts. The results showed that antifungal compounds were produced by *Streptomyces* sp. strain SO6 using PW as a nutrient source and released into the culture medium. Fig 4a shows the mycelial growth inhibition (19 mm in diameter) of the plant pathogen *B*. *cinerea* by the cell-free supernatant EtOAc extract at a concentration of 100 μg per disc. In contrast, no inhibition of *B*. *cinerea* was observed in the negative control treatment (EtOAc) (Fig 4a).

An absence of the typical orange-red coloration was also observed in the cell-free supernatant after separating it from the biomass. Although the biomass EtOAc extract showed no antifungal activity, a marked orange-red coloration was observed (Fig 4a). From these results, the studies were directed to analyze the antifungal compounds in the cell-free supernatant EtOAc extract, while biopigments were analyzed in the biomass EtOAc extract. For this, the compounds in both the cell-free supernatant and the biomass EtOAc extracts were fractionated separately by means of CC and TLC. Fractions with similar TLC patterns were pooled to give TLC homogeneous groups and determine the yields of antifungals and biopigments.

Among the 15 homogeneous groups of fractions (F1 to F15) from the cell-free supernatant EtOAc extract, two groups showed high antifungal activity against *B*. *cinerea* growth (Fig 4b). Groups F7 and F8 (28 mg total yield) showed inhibition halos of 9 ± 1 and 19 ± 3 mm (in diameter), respectively.

**Fig 5:**
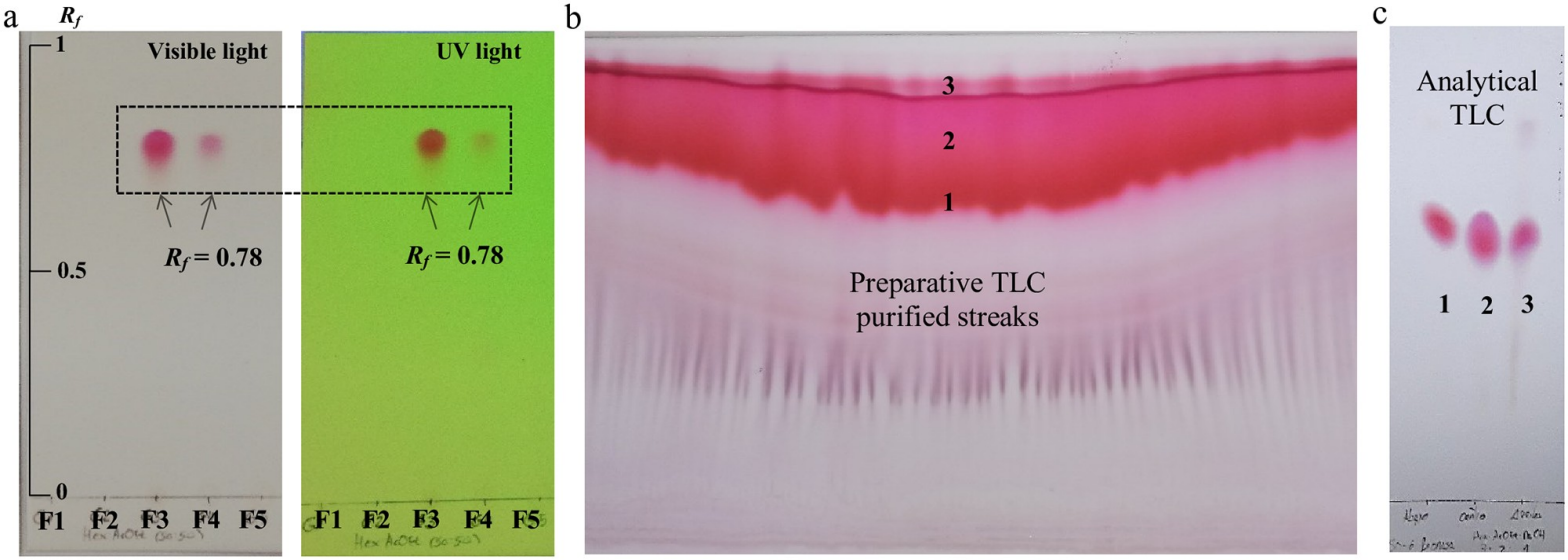
Thin-layer chromatography (TLC) plates showing migration of the reddish-purple biopigments found in biomass EtOAc extracts. Analytical TLC plate of F1 to F5 groups (mobile phase Hex:EtOAc (50:50)) visualized under visible light (left) and 254 nm UV lamp (right) (a); preparative TLC plate of mixed F3 and F4 groups (mobile phase Hex:EtOAc:MeOH (7:2:1)) (b); purification of biopigments using preparative TLC (mobile phase Hex:EtOAc:MeOH (7:2:1)) (c).

A total of 12 homogenous groups were also obtained from the biomass EtOAc extract by combining fractions with identical TLC patterns. Groups F3 and F4 were pooled later due to their identical coloration and *Rf* values, obtaining a total yield of 15.3 mg of a reddish-purple pigment (Fig 5a). The preparative TLC showed at least three major reddish-purple bands (Fig 5b). Each band was collected and concentrated, yielding 1.3 mg (1), 2.1 mg (2), and 1.8 mg (3). One of the obtained bands showed a high purity since other bands were not observed under visible and UV light (Fig 5c).

## Discussion

Actinobacteria can be used in the bioconversion of agricultural wastes into high-value chemical products. The bioconversion of these wastes is catalyzed by a number of enzymes which are responsible for waste degradation [1]. The *Streptomyces* genus has been particularly highlighted because its species possess the potential to secrete broad range of enzymes, which could be involved in the production of antibiotics and biopigments [46,47]. Therefore, enzymatic characterization with ApiCoryne stripes allows us to identify the production of different enzymes in an easy and fast way. In our study, *Streptomyces* sp. strains SO6, PM1, and PM7 presented a high biochemical activity, being positive for at least 12 of the 13 enzymes tested. Moreover, the three preselected strains were capable of fermenting sugars and showed proteolytic and amylolytic activity, with the latter related with the conversion of starch into shorter polymers of glucose units [48]. The biochemical characteristics of *Streptomyces* spp. SO6 and PM7 granted them the potential to bioconvert starch-rich wastes into interesting bioactive chemicals, including antifungals and biopigments. In our study, we observed different behaviors among the tested *Streptomyces* strains, where strain SO6 stood out due to its ability to maintain antifungal activity against the three target fungi and produce biopigment in PW medium.

Numerous works reported the production of bioactive compounds by Actinobacteria strains using various commercial media, such as ISP2–7 [43,44]. The replacement of these frequently used commercial media with available and low-cost agro-industrial wastes is an advantage for the sustainable industrial production of antibiotics and biopigments. The *Streptomyces* genus has a complex development and differentiation, which can adapt to complex and changing environments by ingenious regulation while producing characteristic secondary metabolites [49]. In our study, a noticeable change in pH of the culture media was observed during the fermentation assay using PW broth and ISP2 medium. When *Streptomyces* SO6 was grown in PW medium, the pH increased to 8 on the second day of incubation, which could favor the degradation of the PW and the release of reducing sugars [50]. The pH increase can be attributable to the assimilation of carbon sources other than starch, with the subsequent release of alkaline byproducts [51]. The release of reducing sugars also coincided with the increase in total proteins on the third day of incubation, reflecting not only the level of extractable proteins originating from the bacterial biomass and PW but also secreted enzymes [52]. Thus, the amylolytic and proteolytic activities of *Streptomyces* sp. SO6 could also be involved in PW solubilization and, therefore, in the increase in reducing sugars and the decrease in total dry weight. In this way, the solubilization of PW and its colonization by *Streptomyces* SO6 hindered the determination of the growth rate in the PW medium. An alternative to evaluate bacterial growth rate is the use of agro-industrial waste suspensions as substrate prepared by filtration or boiling [33]. However, a quantity of waste could be generated after filtration, generating another disposal waste problem if it is not used in subsequent nutrient recovery processes.

The differences obtained in the synthesis of antifungals and biopigments shown by *Streptomyces* spp. in ISP2 and PW media could be associated with differences in microbial growth rates [53] and/or the components of the nutritional support [24]. The PW is composed of damaged potato tubers, which have a high starch content between 60% and 70% on a dry basis [54]. Among the carbon sources, starch is one of the most effective nutritional supplements for increasing antibiotic production [24]. Therefore, its nutritional composition makes PW a rich source of starch for the synthesis of different antibiotic compounds. The pH, temperature, and supplement addition are also relevant parameters that can affect the antibiotic production. Yang and Ling [55] reported optimal conditions to synthetize a high concentration of antibiotics by *Streptomyces viridifaciens* using potato as basal medium. These optimal conditions consisted of an initial pH between 5.8 and 6.0, supplement addition, temperature (26°C), and incubation time (5 days). Some of these conditions were similar to those considered in our study, where the synthesis and release of diffusible antifungals against *B*. *cinerea* were observed. Antibiotics synthesized by *Streptomyces* spp. against *B*. *cinerea* were also reported by Li et al. [16], using wheat seeds as nutritional support to produce and release volatile antifungal compounds. *Streptomyces* spp. have also been able to produce antibiotics (oxytetracycline) in agricultural wastes with high protein and fiber contents such as groundnut shells [22] and agro-industrial wastes supplemented with starch [23].

The microbial pigment production is also related to the hydrolysis of starch. Long et al. [25] indicated that carbohydrate metabolism can contribute to the supply of metabolic flux for acetyl-CoA formation and the expression of key genes responsible for microbial biopigment production. In our study, the magenta biopigment formation coincided with PW degradation, evidenced by an increment in reducing sugars and total protein content after the fourth day of PW fermentation. The absence of pigment formation in ISP2 broth during submerged fermentation could be explained by the culture composition since most biopigments are secreted under stress conditions [56]. Similar bacterial magenta pigments have been extracted using different organic solvents [57,58]. A pink/red-like pigment (λ_max_ = 534 nm) produced by *Streptomyces coelicoflavus* was reported by Assia et al. [57]. According to the authors, the spectral characteristics of these pigments are consistent with nonantibiotic hydrophobic anthracyclines with intracellular localization and photosensitivity. Ramesh et al. [59] also reported an antibacterial red pigment (prodiginine) produced by a *Streptomyces* sp. in commercial culture media (λ_max_ = 528 nm). These studies highlight the potential of *Streptomyces* genus to offer a wide range of biopigments to substitute the synthetic pigments. Despite the great versatility of the *Streptomyces* genus for producing bioactive biopigments in different commercial culture media, there are no recent reports on the use of agro-industrial wastes as basal nutrient support for producing *Streptomyces* pigments.

In addition to the importance of identifying *Streptomyces* strains for producing high-value bioproducts using agro-industrial wastes as substrate, the extraction method is determinant to achieve high efficiency in isolation and environmental sustainability. In our study, *Streptomyces* antifungals and biopigments were extracted using EtOAc from cell-free supernatant and biomass, respectively. EtOAc is commonly reported as an efficient solvent to extracts antibiotics from microbial culture filtrates, exhibiting maximum inhibitory zones when compared with extracts obtained using other organic solvents [43]. This solvent is also considered a green solvent recommended to identify new bioactive compounds, allowing the development of sustainable extraction methods and a reduction in the environmental impact of the chemical extraction process [60,61].

## Conclusion

The three *Streptomyces* sp. strains PM1, PM7, and SO6 exhibited interesting antifungal activity and biopigment production. The *Streptomyces* sp. strain SO6 stood out for its ability to use potato waste for producing both antifungals against the economically important phytopathogen *B*. *cinerea* and intracellular biopigments with reddish-purple coloration. *Streptomyces* pigments were produced after the third day of submerged fermentation in potato waste, when total protein and reducing sugar contents began to increase. Therefore, the biopigment production could be the result of different extracellular enzymes released during the fermentation process. Our finding led to the conclusion that potato waste is a valuable source of nutrients for the simultaneous production of antifungals and biopigments by *Streptomyces* spp. Studies on the chemical characterization and optimization of culture parameters during potato waste fermentation are in progress to facilitate scale-up of the antifungal and biopigment production with different biotechnological applications.

## Supporting information

S1 Fig Chilean potato production and potato wastes generated during potato harvest and processing of potato byproducts. Data obtained from ODEPA [32]. *Losses estimation reported by Torres et al. [27]. Red letters are potato wastes needing valorization strategies. Orange lines indicate new alternatives to valorize discarded potato.
(TIF)

S1 Raw images (PDF)
