# Supplementary figures and images for: Bioconversion of potato solid waste into antifungals and biopigments using Streptomyces spp
Source: PLoS One. 2021 May 21;16(5):e0252113. doi: 10.1371/journal.pone.0252113 (PMC8139487; doi:10.1371/journal.pone.0252113)

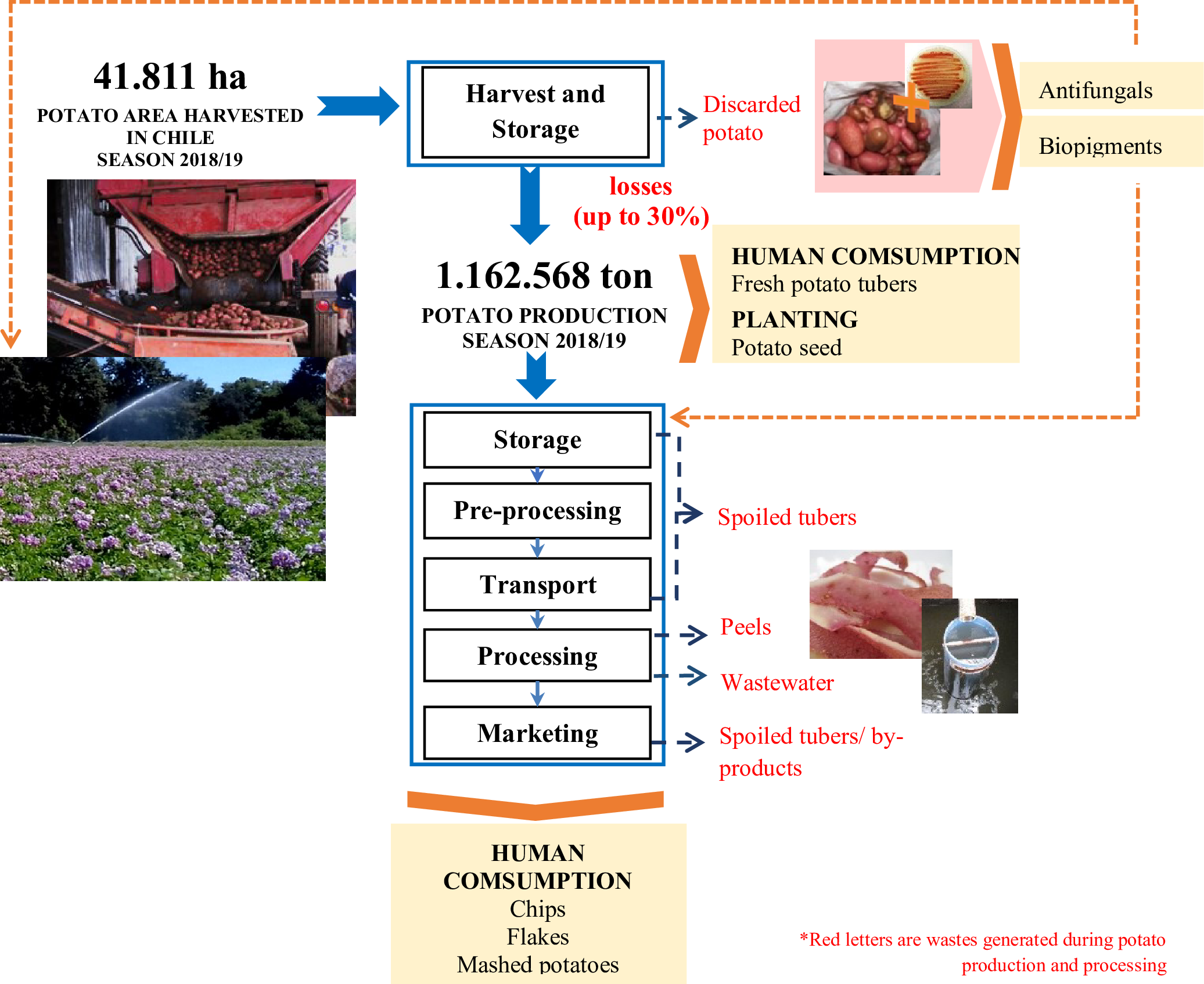

Supplement: S1 Fig — Data obtained from ODEPA [32]. *Losses estimation reported by Torres et al. [27]. Red letters are potato wastes needing valorization strategies. Orange lines indicate new alternatives to valorize discarded potato. (TIF) [file pone.0252113.s001.tif]
