# Supplementary material for: Bioconversion of potato solid waste into antifungals and biopigments using Streptomyces spp
Source: PLoS One. 2021 May 21;16(5):e0252113. doi: 10.1371/journal.pone.0252113 (PMC8139487; doi:10.1371/journal.pone.0252113)

ORIGINAL IMAGES of Thin layer chromatography (TLC) plates composing FIGURE 5

FIG 5 a (TLC plate under visible light)

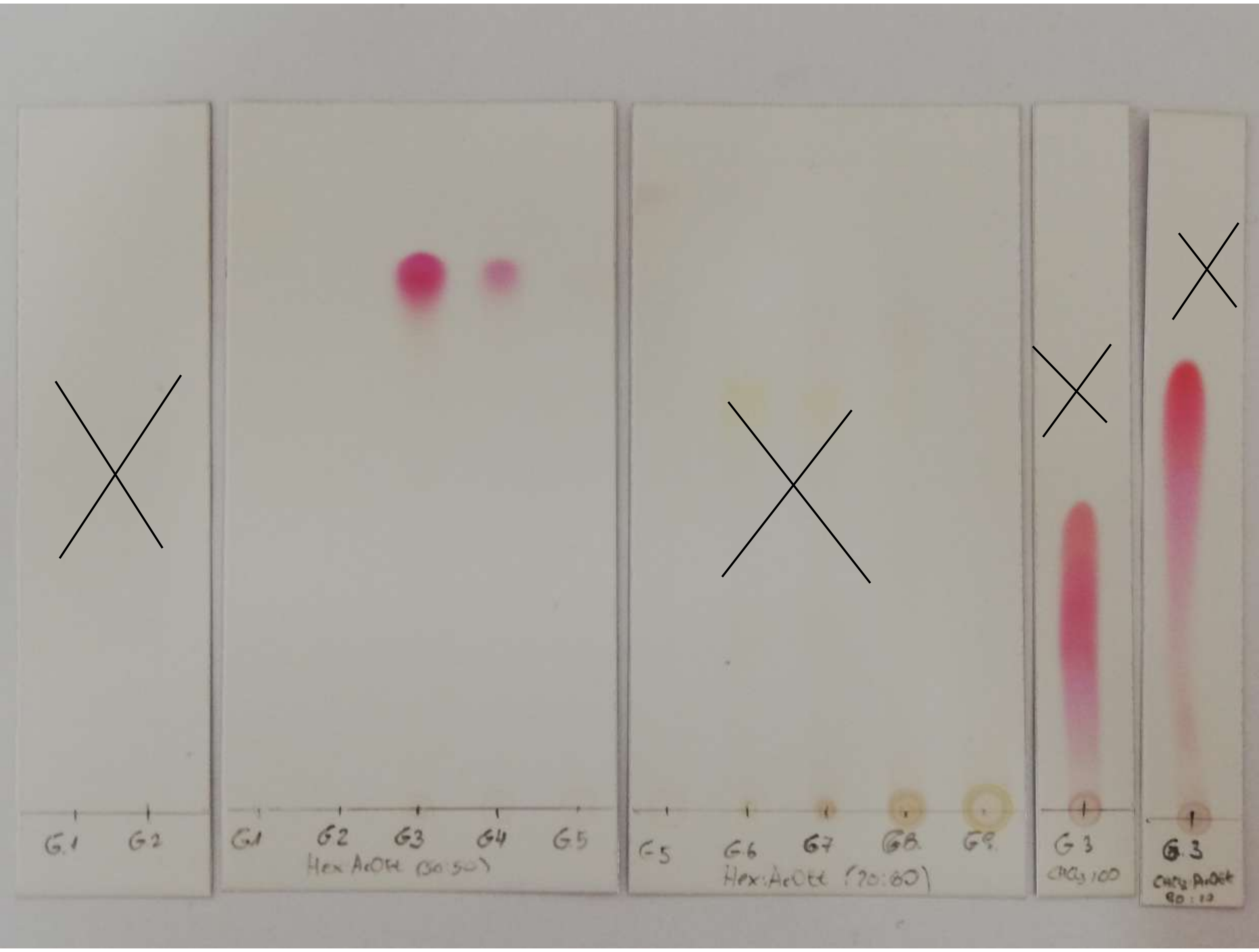

FIG 5 a (TLC plate under UV-light)

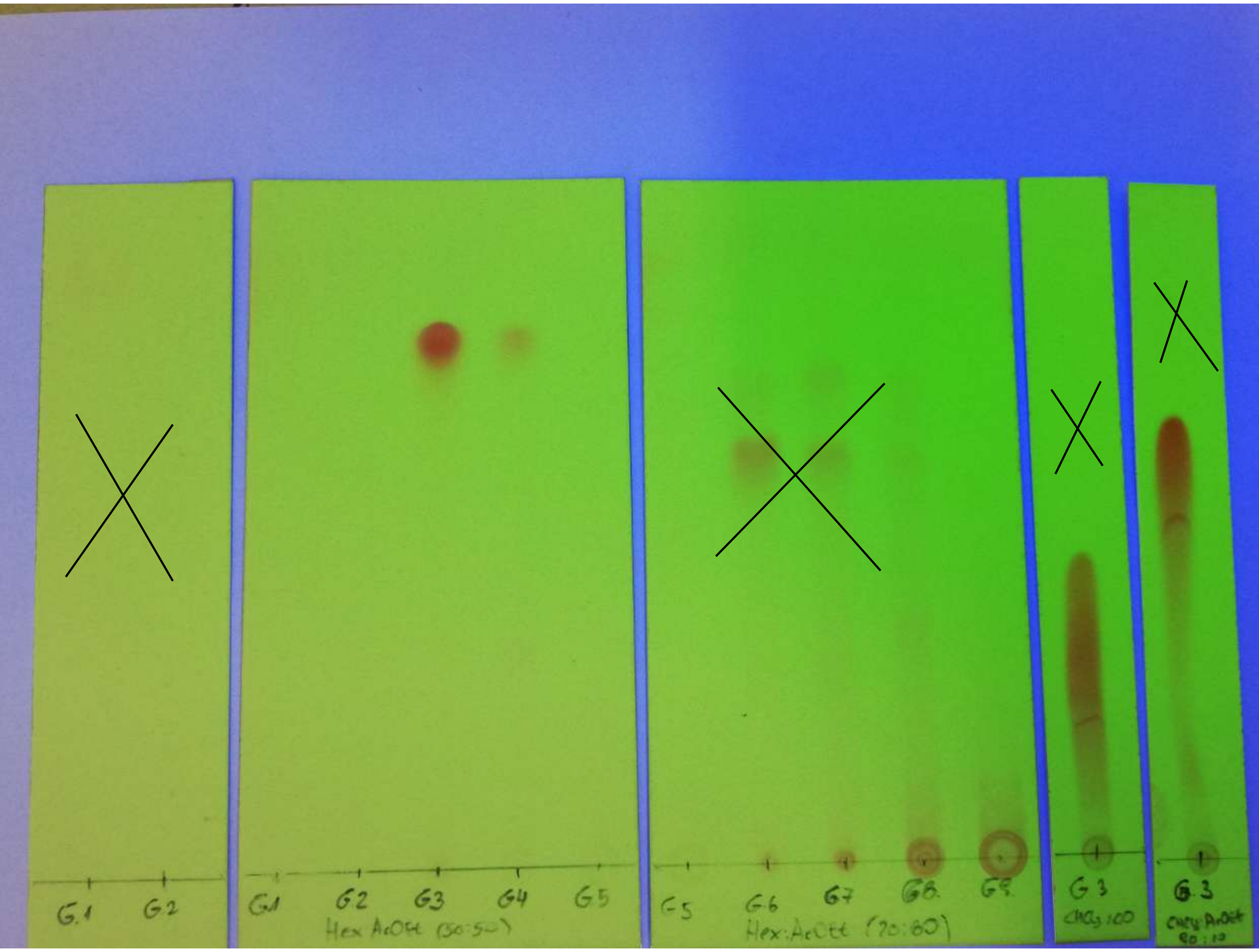

FIG 5 b (preparative TLC plate under visible light)

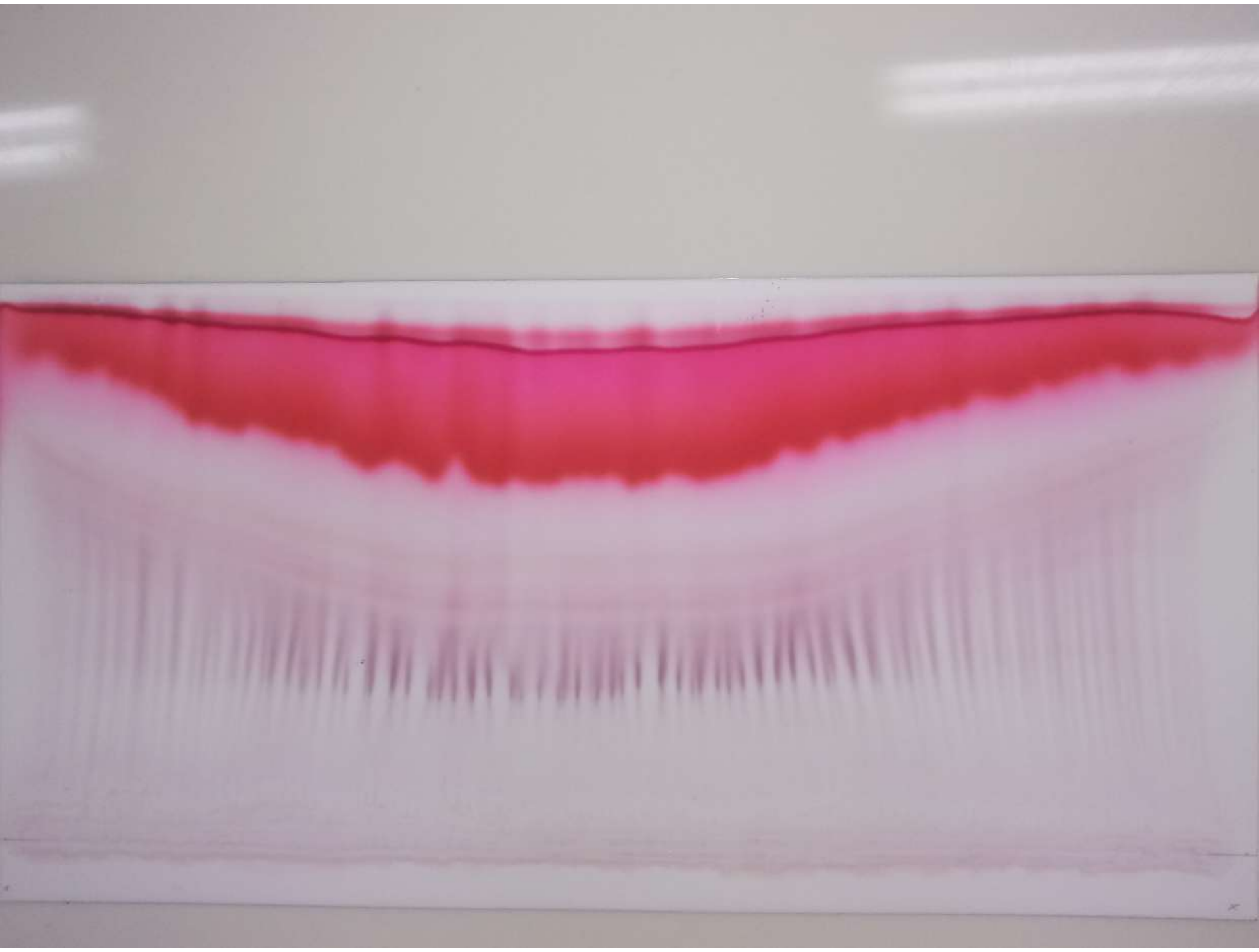

FIG 5 c (Analytical TLC plate under visible light)

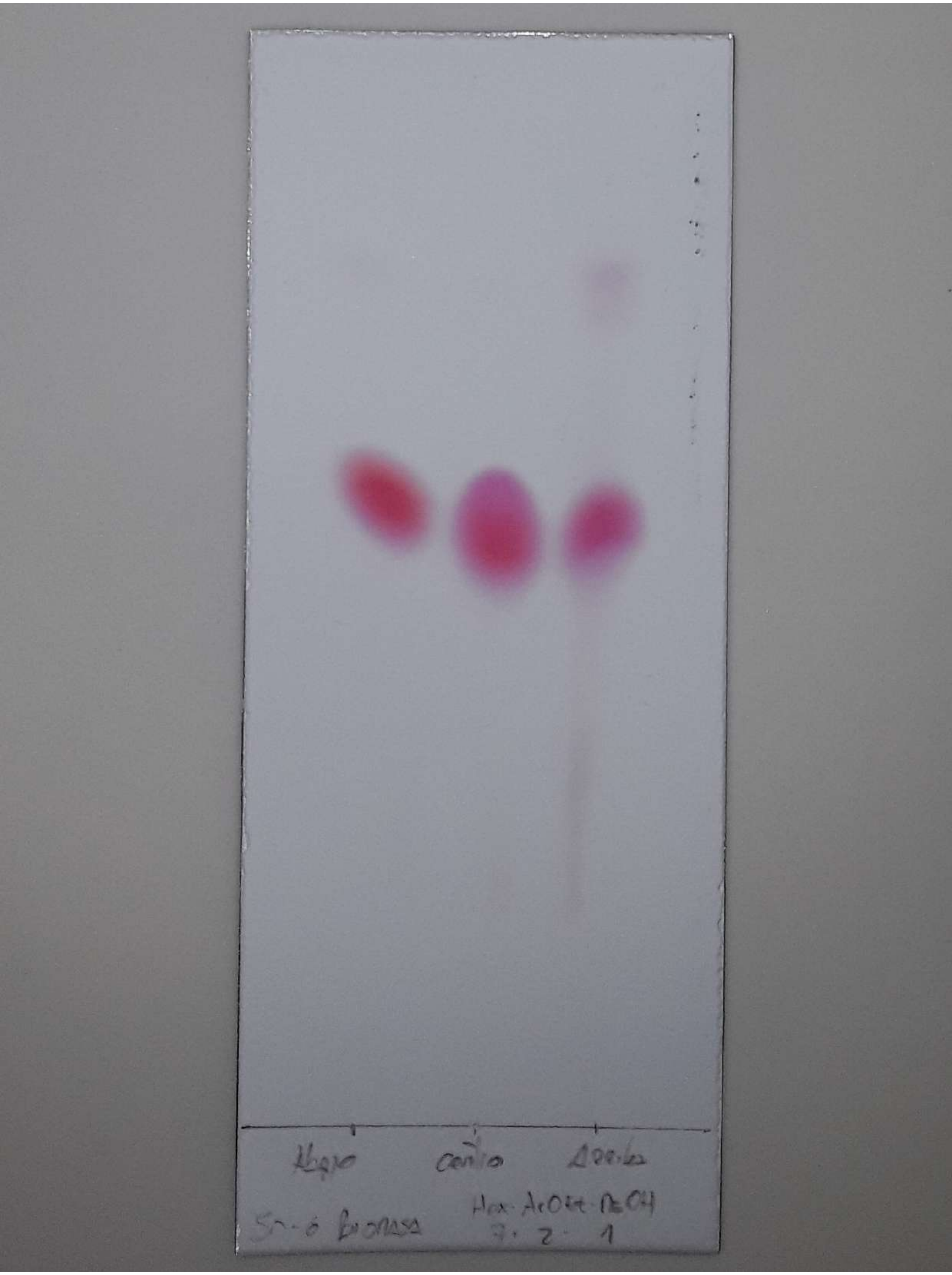

Supplement: S1 Raw images — (PDF) [file pone.0252113.s002.pdf]
